# Forgotten clientele: A systematic review of patient-centered pathology reports

**DOI:** 10.1371/journal.pone.0301116

**Published:** 2024-05-09

**Authors:** Eric Steimetz, Elmira Mostafidi, Carolina Castagna, Raavi Gupta, Rosemary Frasso

**Affiliations:** 1 Department of Pathology, SUNY Downstate Health Sciences University, Brooklyn, New York, United States of America; 2 College of Population Health, Thomas Jefferson University, Philadelphia, Pennsylvania, United States of America; 3 Asano-Gonnella Center for Research in Medical Education and Health Care, Sidney Kimmel Medical College, Philadelphia, Pennsylvania, United States of America; Makere University College of Health Sciences, UGANDA

## Abstract

**Context:**

Patient portals, designed to give ready access to medical records, have led to important improvements in patient care. However, there is a downside: much of the information available on portals is not designed for lay people. Pathology reports are no exception. Access to complex reports often leaves patients confused, concerned and stressed. We conducted a systematic review to explore recommendations and guidelines designed to promote a patient centered approach to pathology reporting.

**Design:**

In consultation with a research librarian, a search strategy was developed to identify literature regarding patient-centered pathology reports (PCPR). Terms such as “pathology reports,” “patient-centered,” and “lay-terms” were used. The PubMed, Embase and Scopus databases were searched during the first quarter of 2023. Studies were included if they were original research and in English, without date restrictions.

**Results:**

Of 1,053 articles identified, 17 underwent a full-text review. Only 5 studies (≈0.5%) met eligibility criteria: two randomized trials; two qualitative studies; a patient survey of perceived utility of potential interventions. A major theme that emerged from the patient survey/qualitative studies is the need for pathology reports to be in simple, non-medical language. Major themes of the quantitative studies were that patients preferred PCPRs, and patients who received PCPRs knew and recalled their cancer stage/grade better than the control group.

**Conclusion:**

Pathology reports play a vital role in the decision-making process for patient care. Yet, they are beyond the comprehension of most patients. No framework or guidelines exist for generating reports that deploy accessible language. PCPRs should be a focus of future interventions to improve patient care.

## Introduction

With few exceptions, pathologists provide the diagnosis, and in many cases, prognosis, for a wide range of medical conditions. Laboratory tests or biopsies are used to diagnose cancer, diabetes, and kidney disease, which are among the ten leading causes of death in the United States [1]. Over ten billion clinical lab tests are ordered and millions of biopsies are performed annually in the United States [2–5]. Despite the sheer volume and everyday use, pathology reports are written in language that is beyond the comprehension of most Americans [6]. Moreover, even content that is written specifically for patients is often too complicated. An analysis of online patient education material found that the language of most articles was above the recommended national health literacy guidelines [7].

In the United States, the 21st Century Cures Act requires that patients are given access to their medical records without delay [8]. Yet, patients are not content with access alone—they seek to understand and better contextualize their results [9,10]. A number of studies have shown better outcomes when patients are involved in the decision-making process (e.g., choosing to undergo certain test or procedures and taking or stopping medications) [11–13]. Yet they cannot participate without understanding their diagnosis. Patients with low-grade prostate cancer, for example, might be offered radically different treatment options, ranging from active survalliance to surgical removal and radiation therapy. However, without proper context it is difficult for patients to make an informed decision about their care.

Recognizing the impact of clear communication on patients’ well-being drove the Mammography Quality Standards Act of 1994, which requires that mammography results sent to patients are written in lay terms [14]. Mammography reports written at a sixth-grade reading level were correlated with higher rates of patient comprehension, engagement, and timely follow-up [15]. Although not mandated by law, progress has been made in radiology, another field that is primarily diagnostic, to develop patient-centered reports that could be easily understood by the average patient [15–17]. The purpose of this review is to evaluate studies, recommendations and guidelines that address improving patient access to clear, easily understood pathology reports.

## Methods

### Search strategy

In consultation with an expert librarian, a query with the relevant keywords and search criteria was developed. Terms such as “pathology reports,” “patient-centeredness,” “lay-terms,” “electronic health records,” and “patient portal” were used (for a complete list of search terms, see S1 Table). Using the Preferred Reporting Items for Systematic reviews and Meta-Analyses (PRISMA) guidelines [18], we searched the PubMed, Embase and Scopus databases. The search was performed in the first quarter of 2023.

Studies were included if they met the following criteria:

- Original research (excluding review articles, editorials, and commentaries).
- Addressed the interpretation or access to pathology reports by patients or their caretakers.
- Articles have been published in English.

S1 Table contains a copy of the full-length search strategy.

### Selection strategy

**Fig 1:**
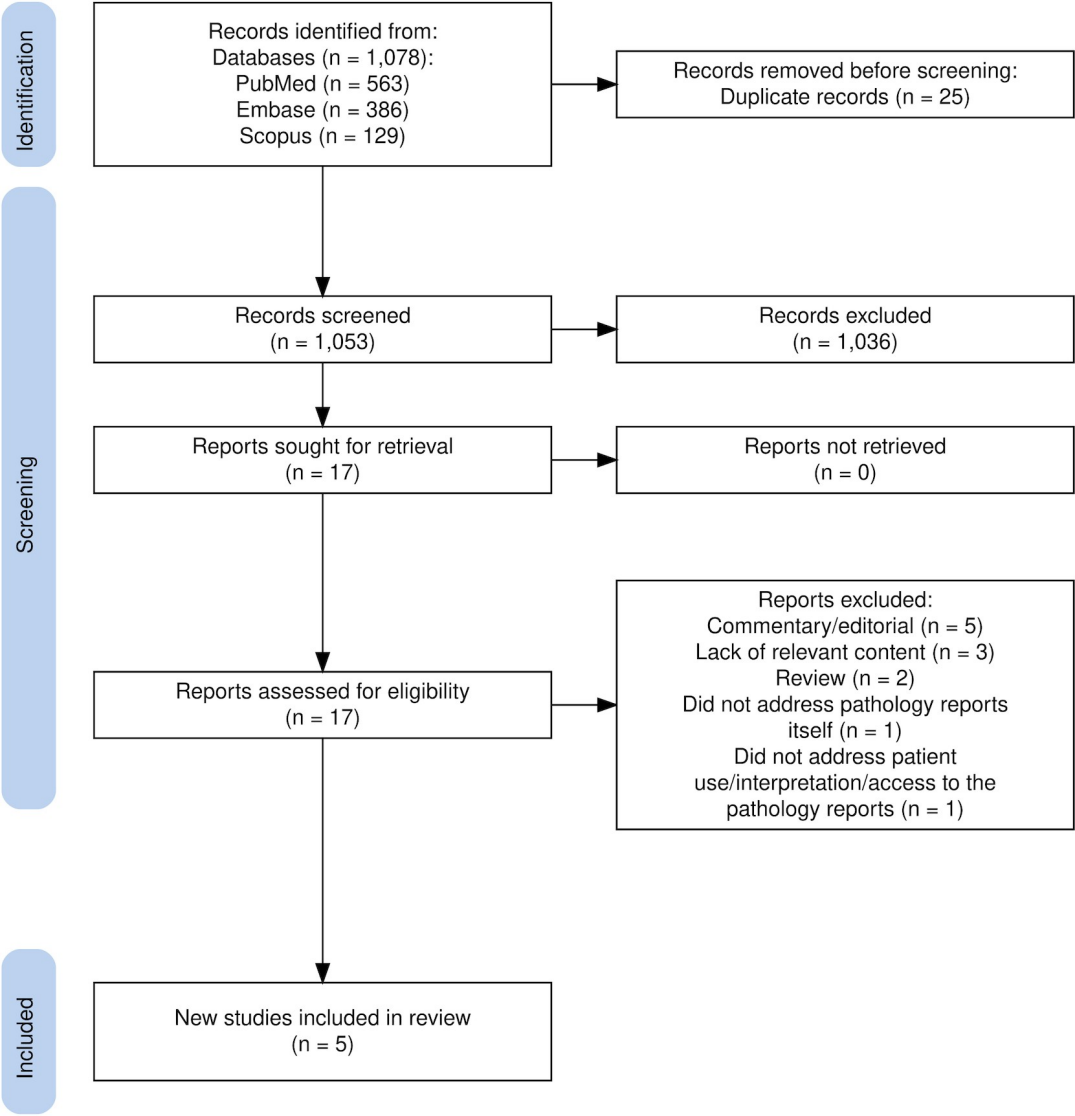
PRISMA flow diagram [18,19].

Two authors (E. S., E. M.) independently reviewed the articles retrieved from the initial search. After eliminating duplicates, the articles were screened by study title and abstract to determine potential relevance to the research question. Articles that met inclusion criteria were selected for full-text review. Discrepancies were resolved through discussion. Next, the authors reviewed full-text articles and independently determined which articles to include in the final analysis. A third author (C.C.) functioned as a tiebreaker in the event of disagreement. The selection process is illustrated in Fig 1.

### Quality assessment

Two researchers (C.C., E.S.) independently conducted the methodological quality assessment of the studies included in the systematic review, based on the different study designs. Disagreements were solved through discussion. The Appraisal tool for Cross-Sectional Studies (AXIS) [20] was used to assess the cross sectional studies; the Critical Appraisal Skills Programme (CASP) tool was used for the qualitative research [21] and the randomized control trials in our analytic sample of papers [22].

The research team organized the papers in the sample into three categories, based on the percent of quality criteria met in each assessment tool: Group 1, studies satisfied at least 75% of the quality criteria, Group 2 between 55–74% and Group 3 less than 55%.

## Results

**Fig 2:**
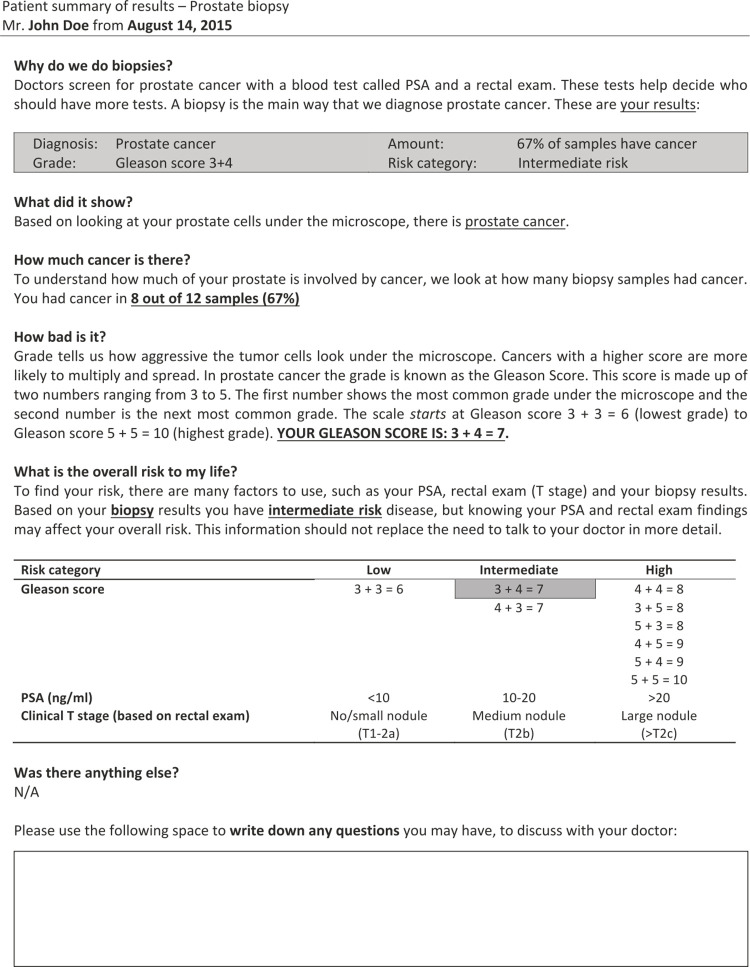
An example of a PCPR [25].

Out of 1,053 potential articles, 17 underwent a full-text review. Of those, 12 articles were excluded due to their study design, commentary/editorial (n = 5); lack of relevant content (n = 3); review (n = 2); they did not address the pathology *report* itself (n = 1); or did not address patient use, interpretation or access to the pathology reports (n = 1). Only 5 (approximately 0.5%) met inclusion criteria. Studies were categorized based on their study design, into qualitative studies/patient surveys and quantitative/randomized controlled trials. The descriptive characteristics of the studies included, along with a summary of relevant findings, appear in Table 1. An example of a PCPR can be found in Fig 2.

**Table 1:** Summary of included studies.

Author, year, citation	Study type	Population	Intervention/Method	Relevant findings
Verosky et al, 2022 [23]	Cross-sectional	Mammography patients	Questionnaire	Patients believe the following would be helpful: • A brief paragraph summarizing important results in nonmedical language • An electronic glossary embedded within the report allowing patients to receive additional information about the terms used • An electronic pamphlet with specific information regarding the results
Austin et al, 2021 [24]	Qualitative	Patients with breast cancer, colorectal cancer or polypectomy	Focus groups	Patients believe that a standard report (SR): • Contains no explanation of the results • Impacts the ability of patients to communicate effectively with providers • Hinders the ability of patients to participate in decision making Patients stated that an ideal PCPR should: • Contain simple language • Highlight information pertinent to decision making • Contain recommended next steps/a roadmap for the future
Nayak et al, 2020 [25]	RCT	Patients with prostate cancer	PCPR	PCPR: • Viewed more favorably than SR • Patients found the language easier to understand • Majority of patients would prefer to receive a PCPR after every biopsy • Facilitates better provider communications • Patients had improved ability to identify and recall important elements of their report (such as Gleason score)
Mossanen et al, 2016 [26]	RCT	Patients undergoing bladder biopsy	PCPR	PCPR: • Patients found the language easier to understand • Majority of patients would prefer to receive a PCPR after every biopsy • Patients had improved ability to identify and recall important elements of their report (such as cancer stage)
Stuckey et al, 2015 [27]	Qualitative	Parents of pediatric patients undergoing whole genome sequencing	Focus groups	A standard report: • Leaves the patient with uncertainty • Provides no information about the next steps PCPR: • Contains language that is easier to understand • Helps facilitate better communication with both physicians and family members • Should contain recommended next steps/what to expect in the future

### Results of quality assessment

According to the summary of the quality assessment performed: one study (Austin et al., 2021) was included in Group 1, since it met 80% of the quality criteria. The rest of the studies were placed into Group 2, as they met between 55 and 74% of the criteria, respectively: Verosky et al., 2022, 73%; Mossanen et al., 2016, 73%; Stuckey et al., 2015, 70%; Nayak et al., 2020, 64%. None of the studies in our sample fell into Group 3. For a detailed breakdown of each study please see S1 File.

### Thematic content/findings

Qualitative review of the final sample of papers yielded the following two key themes:

- Lay Language Use: Using simple, non-medical language needs to be an essential part of a patient-centered report. This was directly addressed in all five studies [23–27]. Authors noted that medical jargon in reports often leaves patients confused and uncertain about their diagnosis. Additionally, the manner in which information is shared directly with patients influences if, and how well, patients can use that information to participate in decisions or actions related to their care [24,27].
- Patient Centered Pathology Reporting (PCPR): Patient-centered pathology reports have the potential to improve communications between providers and patients [25,27], and providers and family members of the patient [27]. Patients who received a PCPR were able to recall important elements from their report, such as the stage or grade of their cancer [25,26]. Most patients who received a PCPR preferred it to a standard report [25,26].

## Discussion

Our systematic exploration of the literature only identified five papers that addressed the importance of patient centered or lay language use in pathology reporting, despite the fact that patients have ready access to these reports. Pathology reports are routinely written in language that is beyond the comprehension of most patients, and studies have shown this is a burden on patients in terms of stress, anxiety and effective management of their conditions [6,7,10]. A systematic review from 2014 noted that not a single paper about pathology reporting discussed *patients* as a target audience or consumer of the report, nor did any paper address the level of health literacy needed for patients to comprehend pathology reports [28]. Despite the importance of clear communication in healthcare, little attention has been paid to the issue of pathology reporting, as evident by the small number of studies we found.

Many studies have linked patient involvement in their care with better outcomes [11–13]. However, patients cannot be part of the decision-making process if the pathology report is beyond their comprehension. This problem is not unique to pathology and is relevant to other diagnostic fields. Much progress has been made in radiology, which much like pathology, is diagnostic in nature [15–17]. While a few changes, such as lay mammography reports, were mandated by law, other initiatives were spurred by recognizing that understanding a diagnostic report is one of the patients’ needs. Although it could be argued that the discipline of pathology is unique in the sense that it is largely devoid of direct clinician-patient interaction, pathologists must recognize that, ultimately, patients are recipients of their reports.

### Potential intervention and other concerns

- The College of American Pathologists provides synoptic cancer reporting templates that are widely used in anatomic pathology [29]. In many instances, those reports are generated electronically based on the information entered by pathologists. It should be feasible to add a lay summary that is automatically generated along with the synoptic report.
- Pathologists should follow the model used in radiology, that is lay reports are shared routinely for mammography and patients are reminded to consult with their clinicians as they make decisions on how to act or not act on findings.
- The introduction of Large Language Models, such as ChatGPT, allows patients to upload their report to the model and request an explanation. However, that response might not be accurate, potentially exacerbating the issue.
- Another solution might be for pathologists to have office hours where they explain the reports to patients. Another benefit of offering this service is allowing physicians to discuss reports with a pathologist and ask for clarification.

### Strengths and limitations

A rigorous search of the literature was conducted, retrieving more than 1,000 articles across three large databases. A third author served as a tiebreaker, to resolve disagreements and minimize potential bias. The overall quality of studies was good, as they met most quality assessment criteria.

Despite a robust search of the literature, only five articles met inclusion criteria for the final analysis. Of those, only two contained quantitative measures, precluding performing a meta-analysis on the results.

## Conclusion

Patient-centered pathology reports are an important topic that has been largely ignored. Despite a rigorous search of the literature, only five studies met inclusion criteria and were included in the final analysis. Although patients have instant access to their pathology reports, no framework or guidelines exist for generating reports with accessible language. Patient-Centered Pathology Reports should be a focus of future interventions to improve patient care.

## Supporting information

S1 Checklist PRISMA checklist. (PDF)

S1 Table Search strategy. (DOCX)

S1 File Quality assessment. (XLSX)
