# Supplementary material for: Forgotten clientele: A systematic review of patient-centered pathology reports
Source: PLoS One. 2024 May 9;19(5):e0301116. doi: 10.1371/journal.pone.0301116 (PMC11081212; doi:10.1371/journal.pone.0301116)
Supplement: S1 Table — (DOCX) [file pone.0301116.s002.docx]

| **Database** | PUBMED | EMBASE | SCOPUS |
| --- | --- | --- | --- |
| **Date** | 1/24/23 | 2/18/23 | 3/24/23 |
| **Strategy** | #1 AND #2 AND #3 | #1 AND #2 AND #3 | #1 AND #2 AND #3 |
| **#1** | "Pathology, Surgical"[Mesh] OR Pathology reports OR Pathology diagnosis OR Pathology record OR "Pathology"[Mesh] OR pathology diagnoses OR "Biopsy"[Mesh] OR Histopathological OR Histopathology | 'Pathology, Surgical'/de OR Pathology reports OR Pathology diagnosis OR Pathology record OR 'Pathology'/de OR pathology diagnoses OR 'Biopsy'/de OR Histopathological OR Histopathology | pathology AND surgical OR pathology AND reports OR pathology AND diagnosis OR pathology AND record OR pathology OR pathology AND diagnoses OR biopsy OR histopathological OR histopathology |
| **#2** | "Patient Navigation"[Mesh] OR "Patients"[Mesh] OR "Patient-Centered Care"[Mesh] OR "patient-centered" OR "Health Communication"[Mesh] OR patient-friendly language OR Layman’s terms OR layman terms OR layman language OR "Layman’s language" OR Lay OR Layman OR "Physician-Patient Relations"[Mesh] | 'Patient Navigation'/de OR 'Patients'/de OR 'Patient-Centered Care'/de OR "patient-centered" OR 'Health Communication'/de OR patient-friendly language OR Layman`s terms OR layman terms OR layman language OR "Layman`s language" OR Lay OR Layman OR 'Physician-Patient Relations'/de | patient AND navigation OR patients OR "patient-centered" AND care OR "patient-centered" OR "Health Communication" OR "patient-friendly" AND language OR layman`s AND terms OR layman AND terms OR layman AND language OR "Layman`s language" OR lay OR layman OR "Physician-Patient Relations" |
| **#3** | "Electronic Health Records"[Mesh] OR EHR OR EMR OR electronic medical records OR "Health Records, Personal"[Mesh] OR "Patient Generated Health Data"[Mesh] OR "Patient Portals"[Mesh] OR "Decision Making"[Mesh] OR decision-making | 'Electronic Health Records'/de OR EHR OR EMR OR electronic medical records OR 'Health Records, Personal'/de OR 'Patient Generated Health Data'/de OR 'Patient Portals'/de OR 'Decision Making'/de OR decision-making | electronic AND health AND records OR ehr OR emr OR electronic AND medical AND records OR health AND records, AND personal OR patient AND generated AND health AND data OR patient AND portals OR decision AND making OR "decision-making" |
